# Exploring the impact of sanguinarine on lamb immunity based on gut microbiota and metabolomics

**DOI:** 10.3389/fvets.2026.1926738

**Published:** 2026-09-10

**Authors:** Meiqi Lin, Jiusi Chen, Aoning Zhen, Juanfeng Li, Dingyun Ma, Sumei Zhang, Longxian Zhang, Senyang Li, Fuchun Jian

**Affiliations:** 1 College of Veterinary Medicine, Henan Agricultural University, Zhengzhou, China; 2 International Joint Research Laboratory for Zoonotic Diseases of Henan, Zhengzhou, China

**Keywords:** arachidonic acid metabolism, gut microbiota, immune regulation, lamb, metabolomics, sanguinarine

## Abstract

**Introduction:**

Sanguinarine (SNG), a benzophenanthridine alkaloid derived from *Macleaya cordata*, exerts beneficial effects in monogastric animals; however, its mechanisms of action in ruminants remain poorly understood. This study aimed to explore whether dietary SNG supplementation improves growth performance and immune function in lambs via modulating gut microbiota and host metabolism.

**Methods:**

Lambs received dietary SNG extract at 200 or 800 mg/kg body weight (equivalent to 4 and 16 mg/kg BW pure SNG, respectively). Growth performance, serum immune-antioxidant indices, gut microbiota, and metabolomics profiles were determined to evaluate the treatment effects.

**Results:**

SNG supplementation significantly increased average daily gain, up-regulated serum IgG, IL-6 and IFN-γ, and down-regulated IL-4 and IL-10. Antioxidant enzyme activities decreased, whereas malondialdehyde concentration was not altered. Microbiota analysis showed reduced *Blautia* abundance and increased *Monoglobus* and *Colidextribacter* across both SNG-treated groups. Metabolomics revealed modified arachidonic acid (AA) metabolism, with elevated lipid mediators 15(S)-HpEPE and 20-hydroxy LTB_4_ in SNG-fed lambs. Integrative analysis identified a potential “*Blautia*-AA-immune” regulatory pattern: high-dose SNG induced a pro-inflammatory Th1-type response, while low-dose SNG sustained immune homeostasis through anti-inflammatory eicosanoids.

**Discussion:**

Our findings indicate that SNG improves growth performance and immune function in lambs by reshaping gut microbiota and reprogramming host lipid metabolism. This work provides mechanistic support for the application of SNG as a functional feed additive for ruminants.

## 1 Introduction

In ruminant production, the proper regulation of immune function is essential for maintaining animal health and optimal growth performance. Under intensive farming conditions, animals are often subjected to nutritional, environmental, and microbial stressors, which can disrupt immune–metabolic homeostasis and lead to inflammation-associated growth suppression and reduced productivity (1). Evidence suggests that phytogenic feed additives alleviate parasitic infections primarily through reshaping the gut microbiome and its metabolic functions, which in turn modulates the host's immune response (2–4). Sanguinarine (SNG), a benzophenanthridine alkaloid derived from *Macleaya cordata*, has been widely reported for its broad-spectrum antimicrobial, anti-inflammatory, and antioxidant properties (5, 6). Previous studies in monogastric animals, such as pigs and chickens, have demonstrated that SNG supplementation can significantly improve growth performance, reduce intestinal pathogen load, and modulate cytokine expression (7–9). However, the underlying mechanisms through which SNG modulates immune and metabolic functions in ruminants are still poorly understood.

The gut microbiota plays a key role in modulating host immunity through its diverse metabolites (10). Metabolites derived from the gut microbiota—such as short-chain fatty acids (SCFAs), bile acids, and lipid mediators—directly influence cytokine secretion and immune cell polarization (11, 12). In particular, the arachidonic acid (AA) metabolic pathway serves as a critical hub linking lipid metabolism and immune responses, generating eicosanoids such as prostaglandins, leukotrienes, and lipoxins that orchestrate both the initiation and resolution of inflammation (13, 14). Consequently, alterations in the gut microbial community may affect host immune homeostasis by modulating AA metabolic signaling.

Therefore, we hypothesized that SNG supplementation could improve growth performance and immune function in lambs by modulating the gut microbiota and its metabolic products. To test this, we performed an integrated analysis of growth performance, serum immune and antioxidant markers, 16S rRNA sequencing of gut microbiota, and UPLC–MS-based metabolomics. The specific objectives were to evaluate the effects of SNG on growth and immune parameters in lambs, identify the key microbial and metabolic signatures responsive to SNG treatment, and establish a mechanistic framework linking microbiota remodeling, metabolite shifts, and immune modulation. This study provides novel mechanistic insights into how plant alkaloids modulate ruminant immunity via the gut microbiota regulation and offers scientific evidence supporting their potential use as functional feed additives.

## 2 Materials and methods

### 2.1 Preparation of SNG extract

SNG extract (containing 2% SNG as the active ingredient) was obtained from Changsha Kunbo Biotechnology Co., Ltd. (Changsha, China). The extract was derived from *Macleaya cordata* and contained SNG as the major active alkaloid component. Before use, the extract was thoroughly mixed with the basal diet to ensure uniform distribution at the designated inclusion levels.

### 2.2 Experimental design

A total of 24 lambs (60 days old) were obtained from the Xuchang Experimental Base of Henan Agricultural University. The animals were randomly assigned to four treatment groups (*n* = 6 per group): CON: basal diet (control); SL: basal diet supplemented with low-dose SNG extract (200 mg/kg body weight, equal to 4 mg/kg BW pure SNG); SH: basal diet supplemented with high-dose SNG extract (800 mg/kg body weight, equal to 16 mg/kg BW pure SNG); DIC: basal diet supplemented with diclazuril (2 mg/kg body weight; positive control) All groups received the same basal diet formulated according to the nutrient requirements of growing lambs (the composition is shown in Table 1). Feed was offered twice daily at 08:00 and 18:00, and clean water was available ad libitum. The experimental period lasted for 28 days, during which all lambs were housed individually under uniform environmental and management conditions. The supplementary dosage of extract was recalculated and adjusted every 2 weeks based on the real-time live weight of each lamb to maintain stable pure sanguinarine intake per kilogram body weight during the whole trial period.

**Table 1:** Composition and nutritional level of basal diet.

Ingredient	Proportion (%)	Nutrient composition	Content level (%)
Peanut Vine	37.50	Dry Matter	89.2
Corn	28.10	Crude Fiber	17.6
Soybean Meal	21.90	Crude Protein	14.3
Wheat Bran	6.25	Crude Fat	2.3
NaHCO3 (Soda)	2.50	Crude Ash	6.1
Premix	1.25	Calcium	1.07
Salt	1.25	Phosphorus	0.38
CaHPO4	1.25	-	-
Total	100.00	-	-

Dry matter content of the diet is a measured value determined by laboratory analysis. Crude protein, crude fiber, crude fat, crude ash, calcium and phosphorus contents are calculated values based on the Chinese Feed Database (2020). The premix used in this trial was commercial 4% growing lamb premix (Beijing Jingzhun Animal Nutrition Co., Ltd.), suitable for lambs aged 7 days to 4 months. According to the product specification, the guaranteed nutrient contents per kilogram premix are: vitamin A (160,000–400,000 IU), vitamin D3 (25,000–95,000 IU), vitamin E ≥ 700 mg, vitamin K ≥ 25 mg, nicotinic acid ≥ 400 mg, Fe (1000–2500 mg), Cu (80–350 mg), Mn (800–2500 mg), Zn (800–25,000 mg), I (12–50 mg), Se (4–12 mg), Co (4–30 mg), biotin ≥ 0.7 mg, Ca (8–28%), total phosphorus (1–8%). The premix also contains inert carrier.

### 2.3 Sample collection and processing

**Figure 1:**
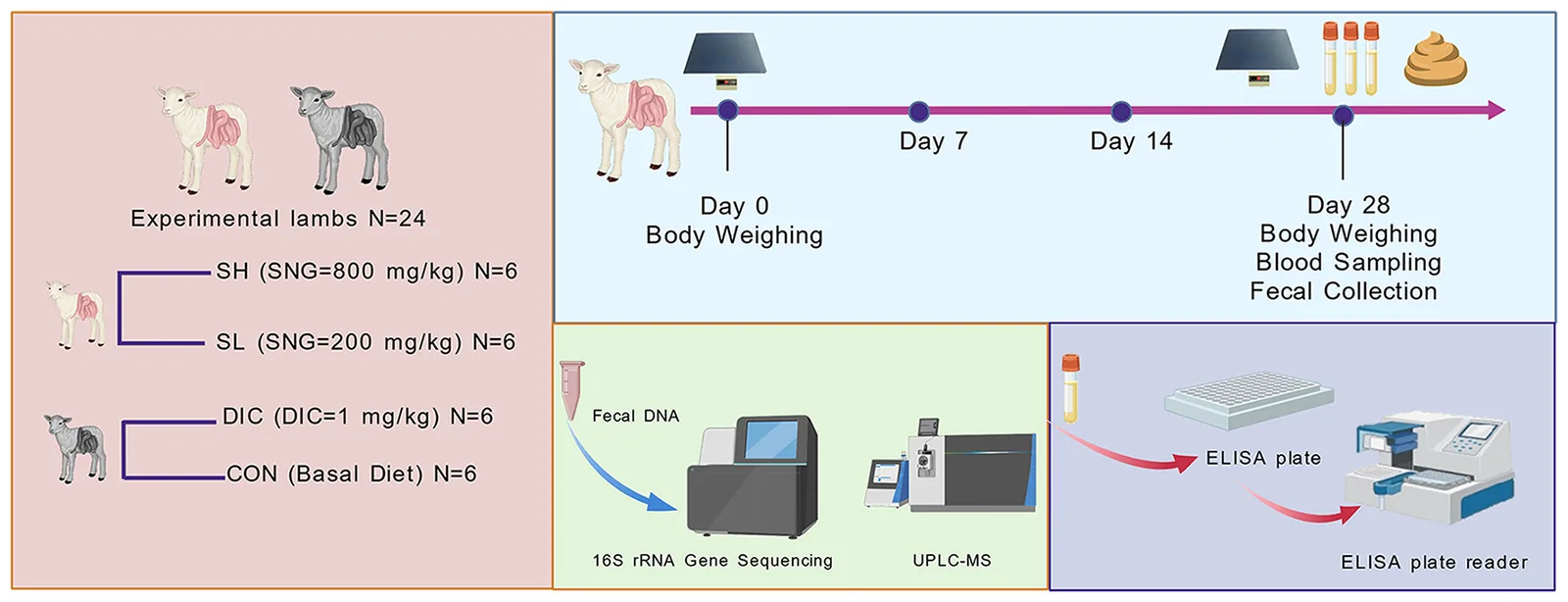
Experimental design and sample collection workflow. SL = 200 mg/kg SNG extract (contains 4 mg/kg BW pure SNG); SH = 800 mg/kg SNG extract (contains 16 mg/kg BW pure SNG). The extract contains 2% w/w pure SNG, and dosage was adjusted biweekly based on individual lamb live weight. *n* = 4 biological replicates selected from 6 lambs per group for omics and serum analyses; all 6 lambs per group were measured for growth performance.

The experimental design, sampling schedule, and analytical workflow are summarized in Figure 1. On days 1 and 28 of the trial, all lambs were weighed in the morning before feeding to record initial body weight (IBW) and final body weight (FBW). The average daily gain (ADG) of each lamb was calculated based on these measurements.


$$\begin{array}{c} \text{ADG }=\;\left(\text{FBW }-\text{ IBW}\right)/28\text{days}. \end{array}$$

On day 28, jugular blood samples (5 mL) and rectal fecal samples were collected from four randomly selected lambs out of six individuals within each of the four groups (CON, DIC, SL, SH; *n* = 4 per group). Blood samples were allowed to clot at room temperature for 30 min and then centrifuged at 3,000 × g for 15 min to obtain serum, which was aliquoted and stored at −20 °C for biochemical assays. Fecal samples were rapidly frozen in liquid nitrogen and stored at −80 °C until gut microbiota sequencing and non-targeted metabolomic analysis. Notably, the same four randomly selected lambs from each group were used for both serum biochemical detection and fecal multi-omics profiling, generating 16 serum samples and 16 fecal samples in total for subsequent analyses. All 24 lambs (*n* = 6 per group) were fully recorded for growth performance indicators including IBW, FBW and ADG.

### 2.4 Serum biochemical analysis

Serum inflammatory, immune, oxidative, and antioxidant parameters were determined using enzyme-linked immunosorbent assay (ELISA) kits (MLbio, Shanghai, China) according to the manufacturer's instructions.The inflammatory markers included interleukin-4 (IL-4), interleukin-6 (IL-6), interleukin-10 (IL-10), interleukin-1β (IL-1β), and interferon-γ (IFN-γ). The immune index included immunoglobulin G (IgG). The oxidative stress parameters included total antioxidant capacity (T-AOC), malondialdehyde (MDA), glutathione peroxidase (GSH-Px), and superoxide dismutase (SOD).

### 2.5 Gut microbiota analysis

A total of 16 fecal samples (four biological replicates randomly chosen from six lambs per group across CON, DIC, SL and SH) were subjected to 16S rRNA gene sequencing. The V3–V4 region of the bacterial 16S rRNA gene was amplified using primers 338F 5′-ACTCCTACGGGAGGCAGCA3′) and 806R 5′-GGACTACHVGGGTWTCTAAT3′). Sequencing libraries were prepared using the TruSeq Nano DNA LT Library Kit (Illumina, USA) and sequenced on the Illumina MiSeq platform (Biozeron Co., Ltd., Shanghai, China). Alpha diversity indices (Chao1, ACE, Shannon, and Simpson) were calculated to estimate within-sample richness and diversity, while beta diversity analysis was performed to compare microbial community similarities among groups. The statistical significance of community differences was assessed by permutational multivariate analysis of variance (PERMANOVA).

### 2.6 Fecal metabolomic analysis

Non-targeted metabolomic profiling of fecal samples was conducted using ultra-performance liquid chromatography–tandem mass spectrometry (UPLC-MS). Briefly, 100 mg of fecal sample (ground in liquid nitrogen) was mixed with 500 μL of pre-cooled 80% methanol, vortexed, and incubated on ice for 5 min. The mixture was centrifuged at 15,000 × g for 20 min at 4 °C, and the supernatant was diluted with MS-grade water to a final methanol concentration of 53%. After a second centrifugation under the same conditions, the supernatant was collected for LC–MS analysis.Principal component analysis (PCA) was used to assess overall metabolic variation, and orthogonal partial least squares discriminant analysis (OPLS-DA) was applied for pattern recognition and identification of differential metabolites based on *p* < 0.05, fold change |log_2_FC| > 1, and variable importance in projection (VIP) > 1. Metabolites were classified according to chemical taxonomy, and pathway enrichment analysis was performed using MetaboAnalyst based on KEGG and Small Molecule Pathway Database (SMPDB) annotations to identify potential biological mechanisms. False discovery rate (FDR) correction was applied where applicable using the Benjamini–Hochberg method.

### 2.7 Statistical analysis

All statistical analyses were performed using SPSS version 25.0 (IBM, USA). Independent-sample *t*-tests were used for pairwise comparisons, and least significant difference (LSD) tests were used for multiple comparisons. Data are expressed as means ± standard error of the mean (SEM). Differences were considered statistically significant at *p* < 0.05. Graphs were generated using GraphPad Prism version 10.1.2 (GraphPad Software, USA).

## 3 Result

### 3.1 Effects of SNG supplementation on growth performance in Hu lambs

As presented in Table 2, IBW differed significantly among groups at the start of the trial (*p* < 0.05), which led to corresponding differences in FBW. To account for this initial variation, ADG was used as the primary measure of growth performance. Dietary supplementation with SNG significantly affected ADG (*p* < 0.05). SH group achieved the greatest ADG (155.71 ± 22.28 g/d), which was significantly higher than that of the CON and DIC groups (*p* < 0.05). The SL group showed an intermediate ADG (128.57 ± 19.33 g/d), not significantly different from the SH group but higher than CON.

**Table 2:** Effects of dietary SNG supplementation on the growth performance of Hu lambs.

Item	CON	DIC	SL (200 mg/kg)	SH (800 mg/kg)
IBW (kg)	24.98 ± 0.74	34.16 ± 2.03	30.24 ± 3.31	25.72 ± 1.66
FBW (kg)	26.94 ± 0.6	36.28 ± 2.06	33.84 ± 2.95	30.08 ± 1.62
ADG (g/d)	75.71 ± 25.64^b^	75.71 ± 29.35^b^	128.57 ± 19.33^ab^	155.71 ± 22.28^a^

SL = 200 mg/kg SNG extract (contains 4 mg/kg BW pure sanguinarine, SNG); SH = 800 mg/kg SNG extract (contains 16 mg/kg BW pure SNG). Different superscript letters within the same row indicate significant differences among groups (*p* < 0.05, LSD *post-hoc* test). Exact pairwise comparison*p*-values for ADG: CON vs. SL, *p* = 0.146; CON vs. SH, *p* = 0.034; CON vs. DIC, *p* = 1.000; SL vs. SH, *p* = 0.444; SL vs. DIC, *p* = 0.146; SH vs. DIC, *p* = 0.034. All comparisons were performed using the LSD test.

### 3.2 Effects of SNG supplementation on serum biochemical and immune parameters

**Figure 2:**
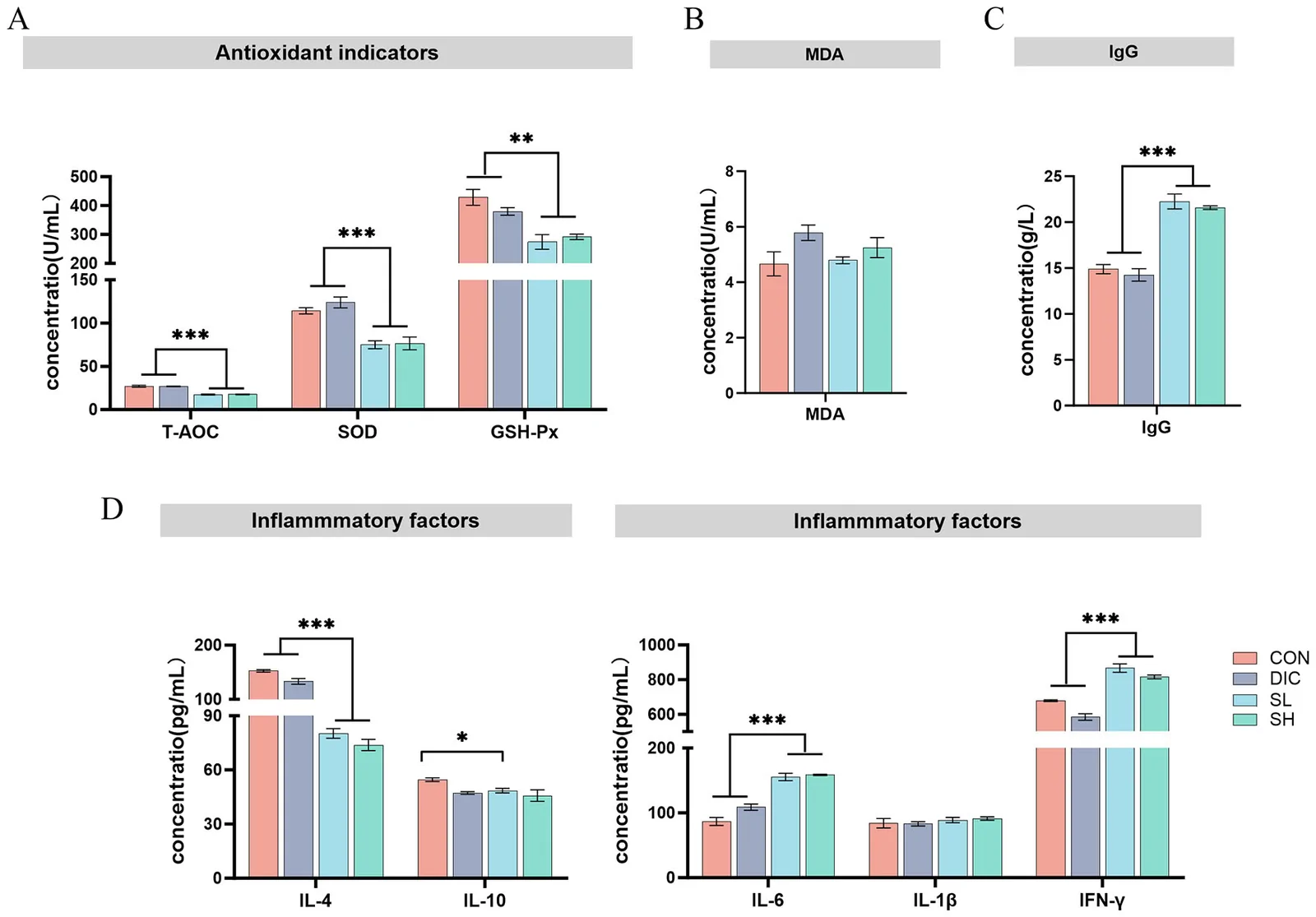
Effects of SNG extract on serum antioxidant, immune, and inflammatory parameters in Hu lambs. **(A)** Antioxidant indicators; **(B)** MDA concentration; **(C)** IgG concentration; **(D)** Inflammatory indicators; Data are presented as mean ± SEM. Asterisks indicate significant differences (*p < 0.05, **p < 0.01, ***p < 0.001). *n* = 4 biological replicates selected from 6 lambs per group.

Serum antioxidant, immune, and inflammatory parameters were analyzed across treatment groups (Table 3; Figure 2). Compared with the control, SNG supplementation significantly reduced the activities of T-AOC, SOD, and GSH-Px in both SL and SH groups (all *p* < 0.001), whereas MDA concentration remained unchanged across all groups. In contrast, serum IgG concentration was significantly elevated in SNG-treated lambs (*p* < 0.001).

**Table 3:** Effects of SNG supplementation on serum antioxidant, immune, and inflammatory parameters in Hu lambs.

Parameter	CON	DIC	SL (200 mg/kg)	SH (800 mg/kg)	p-value (ANOVA)
Antioxidant parameters					
T-AOC (U/mL)	27.17 ± 2.00^a^	26.80 ± 0.66^a^	17.75 ± 1.25^b^	17.72 ± 0.90^b^	<0.001
SOD (U/mL)	114.24 ± 7.19^a^	123.90 ± 12.72^a^	75.04 ± 9.22^b^	76.67 ± 14.77^b^	<0.001
GSH-Px (U/mL)	428.57 ± 54.74^a^	379.96 ± 26.01^a^	274.32 ± 50.13^b^	292.03 ± 18.89^b^	<0.001
MDA (nmol/mL)	4.66 ± 0.87	5.78 ± 0.56	4.79 ± 0.24	5.25 ± 0.72	0.0095
Immune parameter					
IgG (mg/mL)	14.88 ± 1.00^b^	14.25 ± 1.35^b^	22.25 ± 1.63^a^	21.58 ± 0.38^a^	<0.001
Inflammatory cytokines					
IL-4 (pg/mL)	152.60 ± 4.87^a^	133.13 ± 10.17^b^	80.25 ± 5.24^c^	73.83 ± 6.29^c^	<0.001
IL-6 (pg/mL)	86.71 ± 12.26^c^	108.97 ± 9.66^c^	155.37 ± 11.42^a^	158.60 ± 1.82a	<0.001
IL-10 (pg/mL)	54.56 ± 2.02^a^	47.21 ± 1.52^b^	48.55 ± 2.58^b^	45.80 ± 6.39^b^	0.023
IL-1β (pg/mL)	84.01 ± 14.64	83.06 ± 6.48	88.97 ± 8.28	91.31 ± 5.24	0.57
IFN-γ (pg/mL)	677.90 ± 9.72^b^	583.87 ± 38.13^c^	866.50 ± 48.47^a^	815.19 ± 22.60^a^	<0.001

SL = 200 mg/kg SNG extract (contains 4 mg/kg BW pure sanguinarine, SNG); SH = 800 mg/kg SNG extract (contains 16 mg/kg BW pure SNG). Different superscript letters within the same row indicate significant differences among groups (*p* < 0.05). Data are presented as mean ± SEM (*n* = 4 per group). Exact pairwise comparison *p*-values are provided in Supplementary Table S1.

For inflammatory cytokines, SNG supplementation significantly reduced IL-4 levels (*p* < 0.001) while increasing IL-6 and IFN-γ levels (all *p* < 0.001). Moreover, IL-10 was markedly decreased in the SH group (*p* = 0.006), whereas IL-1β showed no significant change across any of the treatment groups (*p* = 0.570). Notably, no significant differences were observed between the DIC and CON groups for most serum immune or antioxidant parameters, except for IL-4 (*p* = 0.002), IL-6 (*p* = 0.007), IL-10 (*p* = 0.015), IFN-γ (*p* = 0.002), and MDA (*p* = 0.030), indicating similar physiological status under normal conditions. Detailed pairwise comparison *p*-values for all parameters are provided in Table 3.

### 3.3 Effects of SNG supplementation on gut microbiota composition in Hu lambs

**Figure 3:**
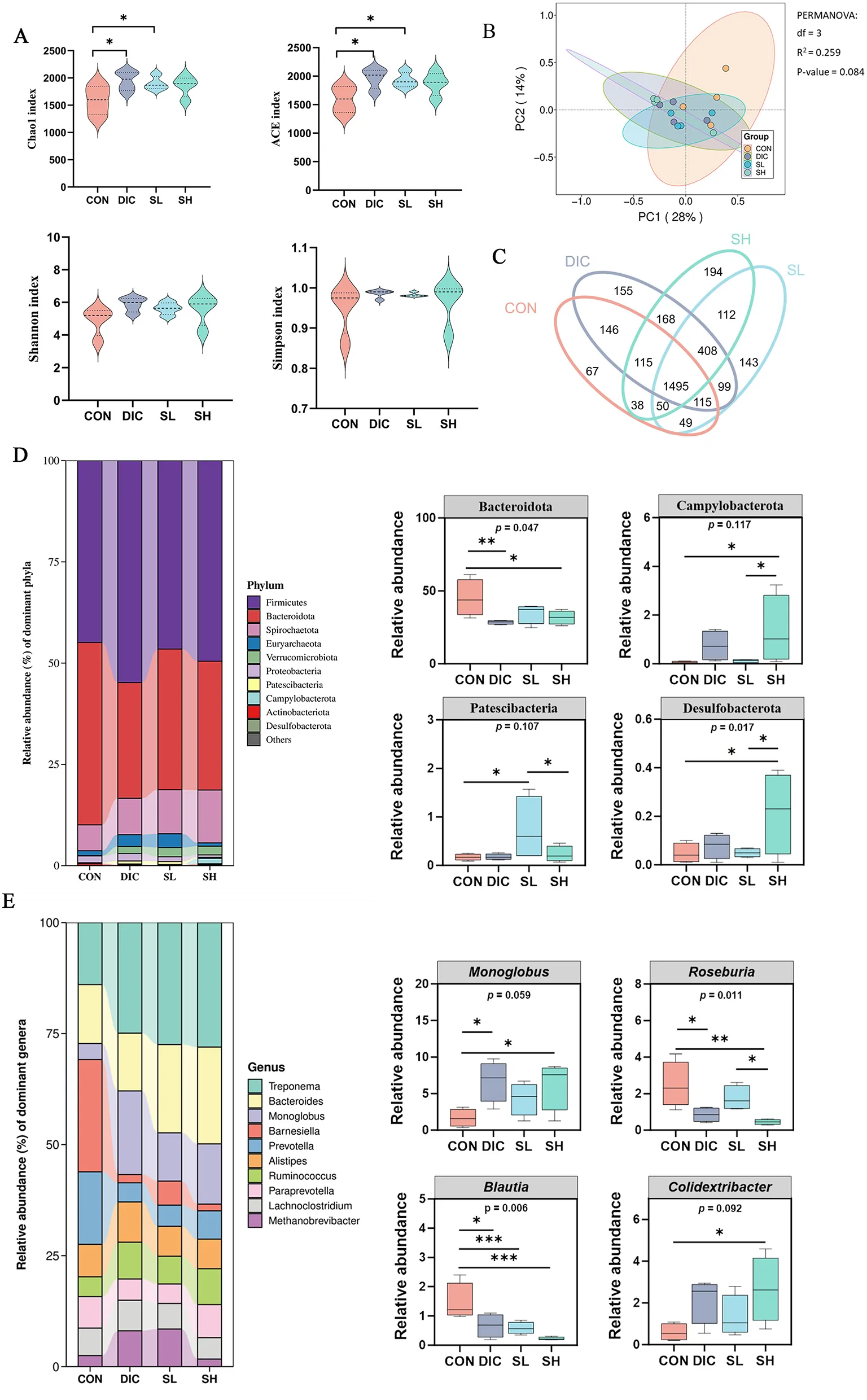
Gut microbial composition of Hu lambs fed SNG extract. **(A)** Alpha diversity indices; **(B)** Beta diversity analysis based on Bray–Curtis distance; **(C)** Relative abundance of dominant phyla across groups; **(D)** Changes in major bacterial phyla; **(E)** Relative abundance of differential genera among groups. Data are presented as mean ± SEM. Asterisks indicate significant differences (*p < 0.05, ** *p* < 0.01, *** *p* < 0.001). *n* = 4 biological replicates selected from 6 lambs per group.

The gut microbiota composition was assessed by 16S rRNA sequencing. Alpha diversity indices (Chao1, ACE, Simpson, and Shannon) were generally elevated in all treatment groups compared with the control, with significantly higher values observed in the SL and DIC groups (*p* < 0.05; Figure 3A). Beta diversity based on Bray–Curtis distance revealed no significant differences in microbial community structure among groups (PERMANOVA: *p* = 0.084, *R*^2^ = 0.259; Figure 3B).

At the phylum level, Firmicutes and Bacteroidota dominated all groups. The relative abundance of Bacteroidota decreased significantly in the DIC and SH groups (*p* < 0.05), while Campylobacterota and Desulfobacterota were enriched specifically in the SH group (*p* < 0.05). In addition, Patescibacteria showed a significant increase exclusively in the SL group (*p* < 0.05; Figure 3D).

At the genus level, *Monoglobus* and *Colidextribacter* displayed increased abundance in all SNG-treated groups, most prominently in SH (*p* < 0.05). In contrast, *Blautia* and *Roseburia* showed decreasing trends in all treated groups, and *Blautia* was significantly reduced in both SL and SH groups (*p* < 0.001; Figure 3E).

### 3.4 Identification of differential microbial taxa by LEfSe and biomarker analysis

**Figure 4:**
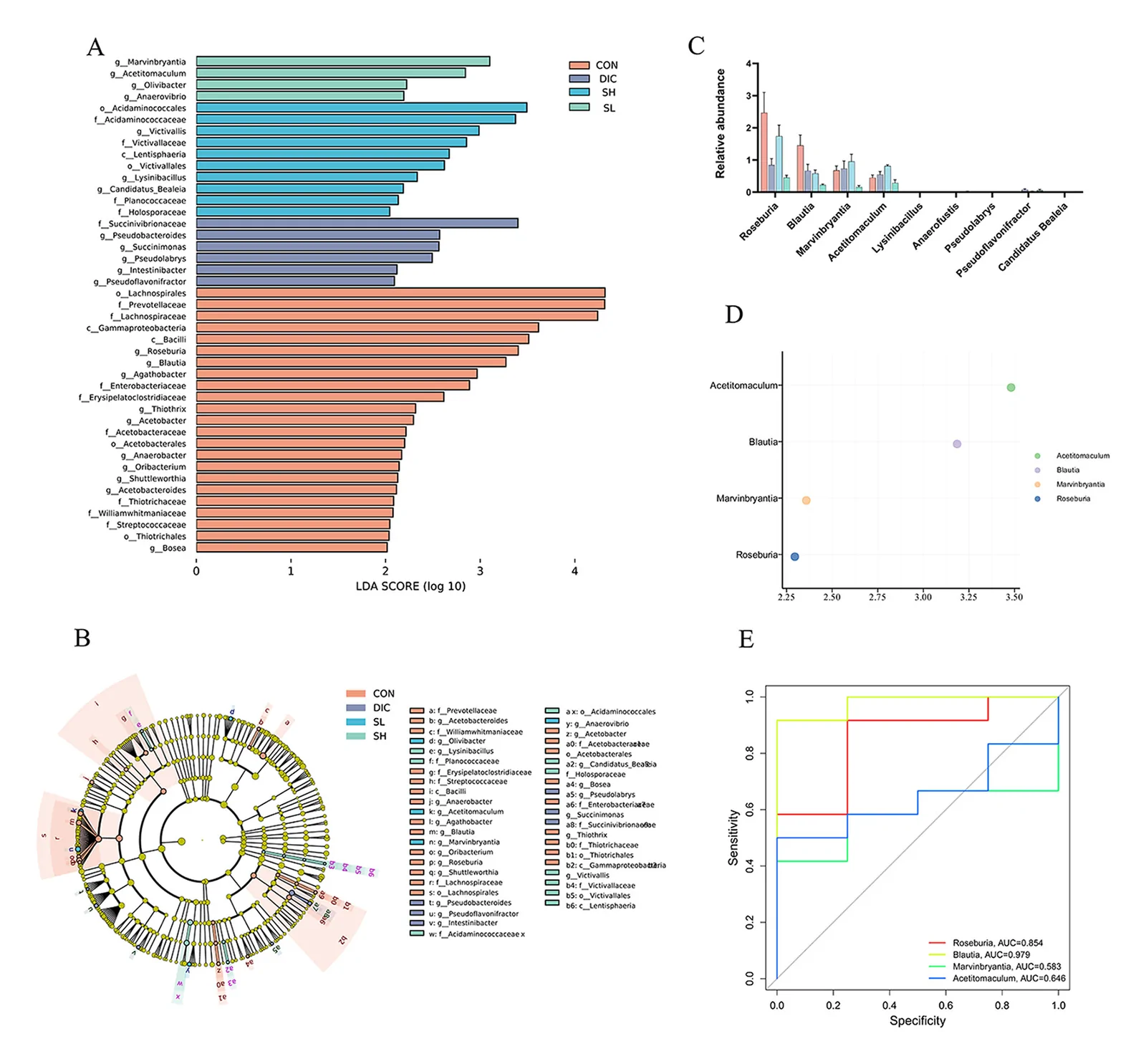
LEfSe and biomarker screening of differential gut bacterial taxa. **(A)** LDA scores; **(B)** Cladogram generated by LEfSe; **(C)** Eight representative differential genera identified; **(D)** Random forest; **(E)** ROC curve. *n* = 4 biological replicates selected from 6 lambs per group.

To identify the microbial taxa most affected by SNG extract supplementation, linear discriminant analysis effect size (LEfSe) was performed with an linear discriminant analysis (LDA) score threshold of 2.0 (*p* < 0.05). This analysis identified 21 differentially abundant bacterial genera across the four experimental groups (Figures 4A, B). *Blautia* and *Roseburia* were significantly enriched in the CON group. Genera including *Marvinbryantia* and *Acetitomaculum* were characteristic biomarkers of the SL group, while taxa such as *Victivallis* and *Lysinibacillus* were enriched in the SH group. *Pseudobacteroides* and *Succinimonas* were discriminative genera for the DIC group. These shifts demonstrate that SNG extract supplementation markedly reshaped gut microbial composition and altered the abundance of genera closely related to host metabolic and immune regulation.

To refine these findings, the two genera with the highest LDA scores from each group were selected as representative taxa. Among the eight selected genera, four with higher relative abundance and consistent responses to SNG supplementation (*Roseburia, Blautia, Marvinbryantia*, and *Acetitomaculum*) were retained for further analysis. Random forest and receiver operating characteristic (ROC) curve analyses were then used to assess their predictive importance. *Blautia* showed the highest feature importance (Gini index = 3.174) and classification accuracy (AUC = 0.979), indicating that it was the most responsive and discriminative genus among the treatment groups.

### 3.5 Functional prediction of gut microbiota based on PICRUSt2

**Figure 5:**
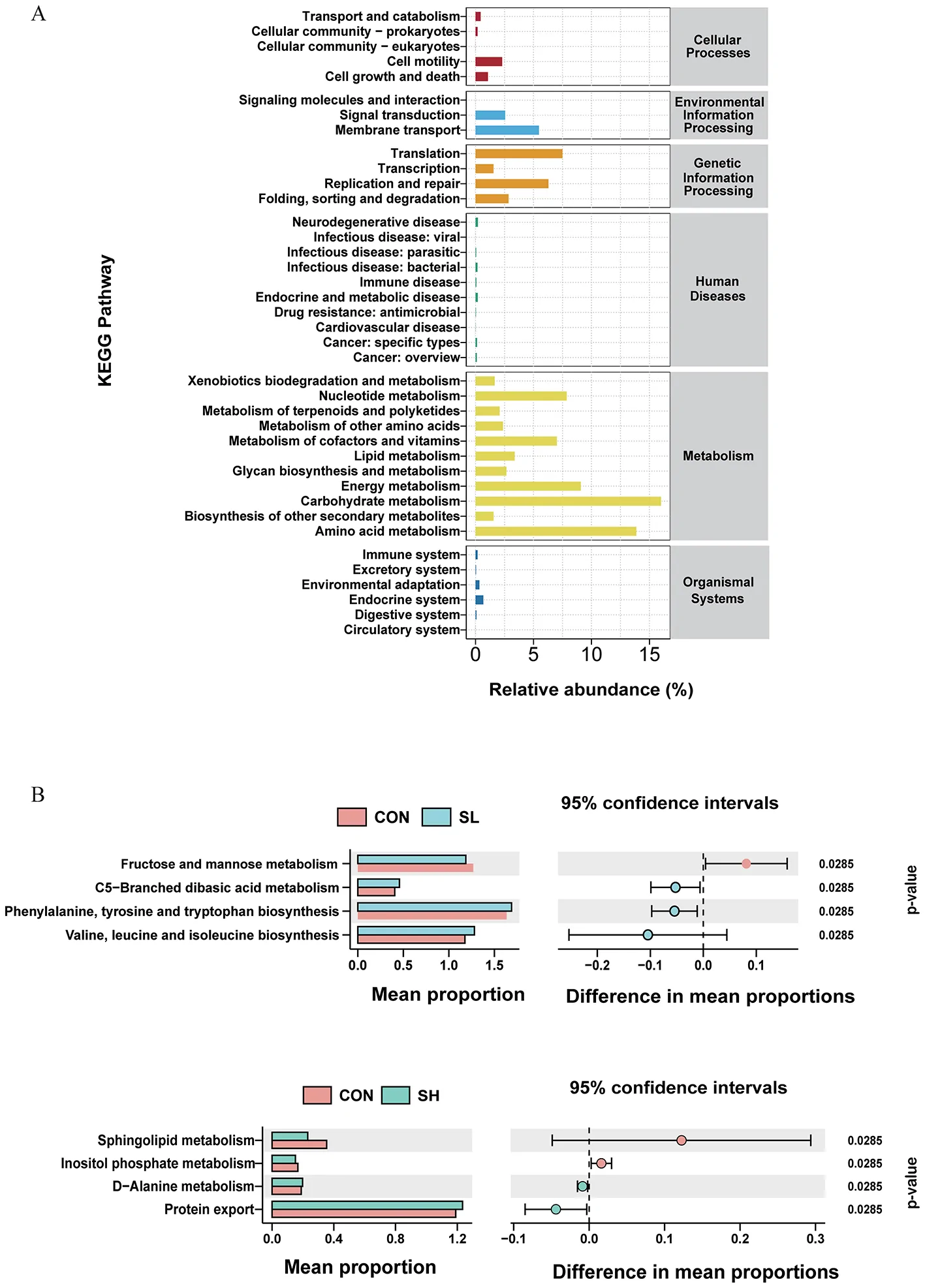
PICRUSt2 predicted functional profiles of gut microbiota. **(A)** KEGG Level 1 function; **(B)** Comparison of KEGG Level 3 pathways. *n* = 4 biological replicates selected from 6 lambs per group.

To explore the potential metabolic capacities influenced by SNG supplementation, we predicted gut microbiota functions using PICRUSt2 based on the KEGG database (Figure 5). At KEGG Level 1, microbial functions were mainly associated with Metabolism, Genetic Information Processing, and Cellular Processes. Level 2 analysis indicated that most predicted pathways were related to amino acid metabolism and carbohydrate metabolism. Further examination at Level 3 revealed distinct functional alterations among treatment groups. Compared with the CON group, the SL group showed significantly increased bacterial abundances associated with fructose and mannose metabolism (*p* < 0.05) but decreased pathways involved in aromatic and branched-chain amino acid biosynthesis (*p* < 0.05). The SH group exhibited significant enrichment in sphingolipid metabolism and phosphatidylinositol signaling system (*p* < 0.05), while D-alanine metabolism and protein export pathways were markedly reduced (*p* < 0.05). These findings indicate that SNG supplementation altered the microbial functional potential related to amino acid and lipid metabolism, which may underlie the metabolic alterations further investigated through metabolomic profiling.

### 3.6 Metabolomic profiling of fecal samples

**Figure 6:**
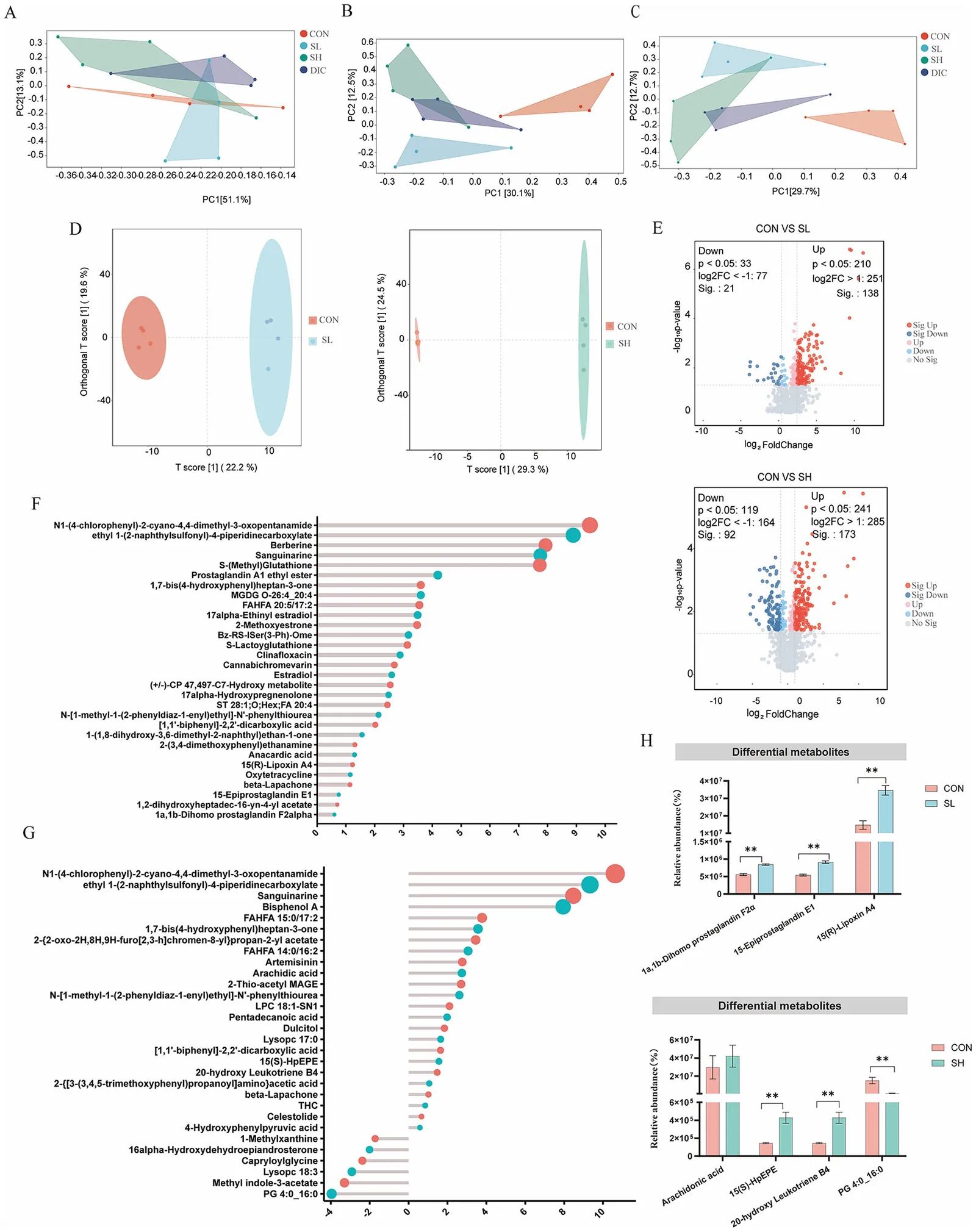
Untargeted fecal metabolomic profiling of lambs supplemented with SNG extract. **(A)** PCA of all samples; **(B)** PCA positive ion mode; **(C)** PCA negative ion mode; **(D)** Pairwise OPLS-DA score plots; **(E )**Volcano plots of differential metabolites; **(F)** CON vs.SL top 30 differential metabolites lollipop map; **(G)** CON vs. SH top 30 differential metabolites lollipop map; **(H)** Differential metabolite abundance. Asterisks indicate significant differences (*p < 0.05, ** *p* < 0.01, *** *p* < 0.001). *n* = 4 biological replicates selected from 6 lambs per group.

The PCA results showed that the samples in each group showed a clear and stable separation trend under the total ion mode (Figure 6A), positive ion mode (Figure 6B) and negative ion mode (Figure 6C). The results showed that SNG supplementation could significantly change the fecal metabolite profile, and there were significant differences in metabolic characteristics among the groups, and the differences were stable under different ion modes. Pairwise OPLS-DA models further demonstrated clear discrimination between the SL and SH groups and the CON group (Figure 6D). Based on the thresholds of |Fold change| > 1, VIP > 1, and *p* < 0.05, a total of 424 differential metabolites were identified across the comparisons. Specifically, 159 differential metabolites (138 upregulated, 21 downregulated) were detected in SL vs. CON, and 265 (173 upregulated, 92 downregulated) in SH vs. CON (Figure 6E). Visualization of the top 30 differential metabolites using a lollipop plot highlighted several key lipid-related metabolites, including prostaglandin E_2_, leukotriene B_4_, and linoleic acid derivatives. These metabolites are involved in AA metabolism and showed higher abundances in the SNG-treated groups compared with the control. Such changes suggested that SNG supplementation may influence lipid oxidation and the metabolic turnover of inflammatory mediators, potentially reflecting an altered immune state in lambs (Figure 6F). Quantitative analysis further confirmed significantly elevated levels of AA-derived compounds in both SL and SH groups relative to CON (Figure 6H).

### 3.7 KEGG pathway enrichment analysis of differential metabolites

**Figure 7:**
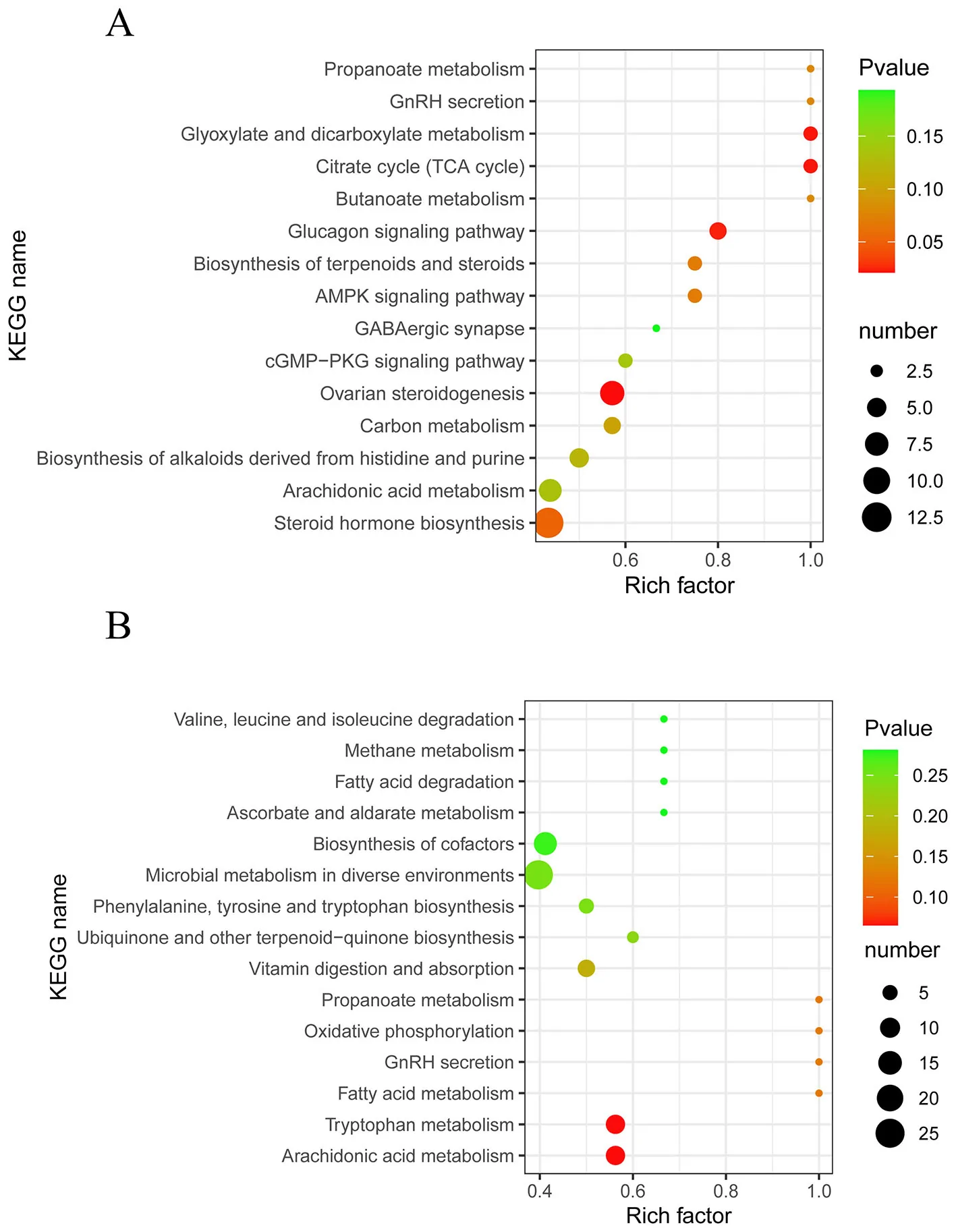
Bubble plots of enriched KEGG metabolic pathways for differential fecal metabolites. **(A)** Enriched KEGG pathways identified in the SL vs. CON comparison; **(B)** Enriched KEGG pathways identified in the SH vs. CON comparison. *n* = 4 biological replicates selected from 6 lambs per group.

To elucidate the biological pathways associated with SNG-induced metabolic changes, KEGG enrichment analysis was performed on the differential metabolites identified in the SL and SH groups (Figure 7). The enriched pathways were mainly related to energy, amino acid, and lipid metabolism. In the SL vs. CON comparison, significant enrichment was observed in ovarian steroidogenesis (*p* = 0.021) and citrate cycle (TCA cycle; *p* = 0.022), while AMPK signaling, propanoate metabolism, and steroid hormone biosynthesis showed enrichment trends (*p* = 0.08). In the SH vs. CON comparison, differential metabolites were primarily mapped to AA metabolism (*p* = 0.065), tryptophan metabolism (*p* = 0.065), and fatty acid metabolism (*p* = 0.122), suggesting that SNG supplementation mainly affected lipid oxidation and inflammatory mediator metabolism.

Notably, the enrichment trend of the AA metabolism pathway showed consistency with the eicosanoid-related differential metabolites identified in Section 3.6, suggesting a potential link between metabolite-level changes and pathway-level trends. Although some enrichments did not reach strict statistical significance, the repeated enrichment trend across both treatment groups suggested that SNG supplementation potentially modulates AA–derived lipid mediator metabolism.

### 3.8 Integrated correlation and mediation analysis of gut microbiota, metabolites, and immune parameters

**Figure 8:**
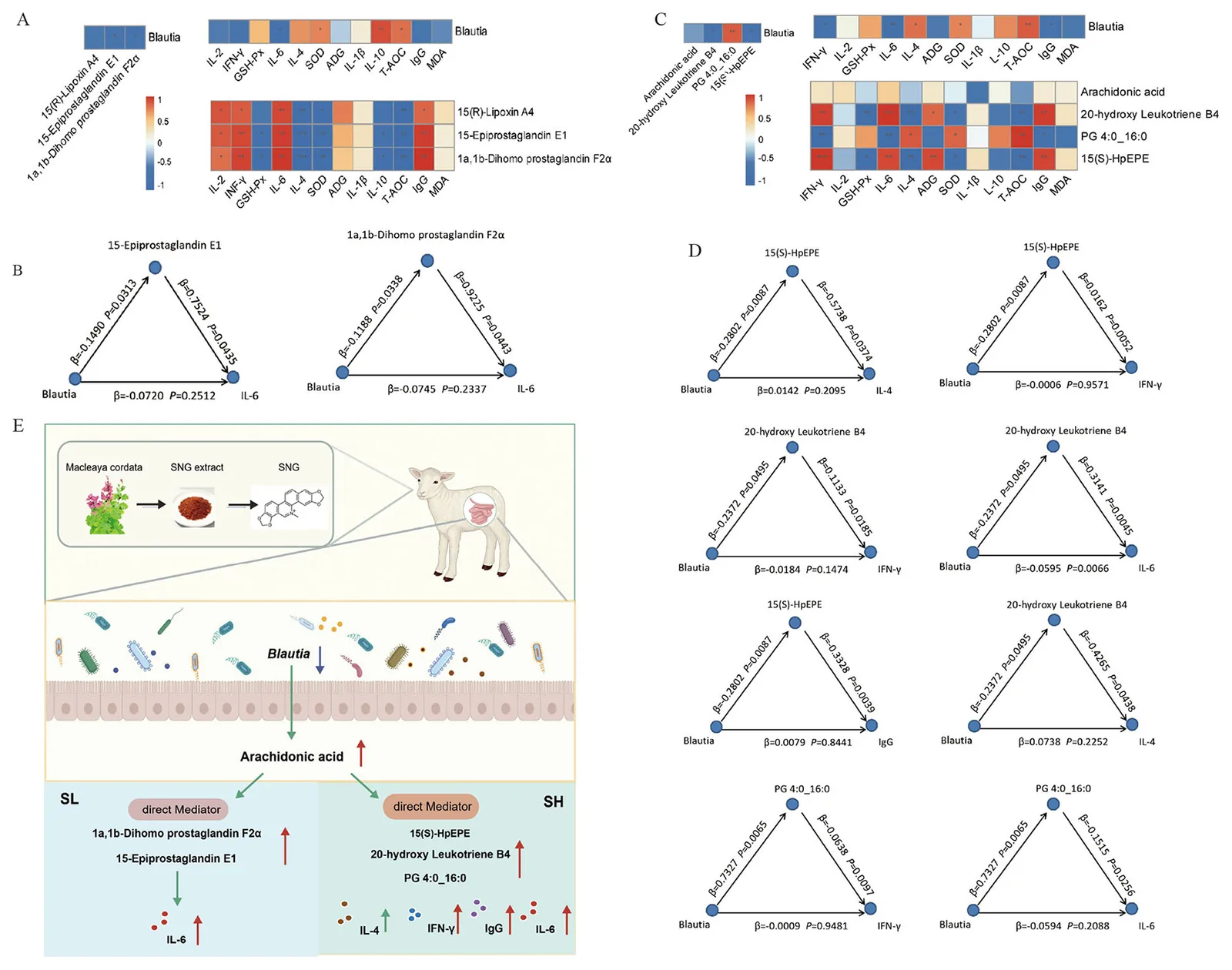
Correlation and mediation analysis linking Blautia, arachidonic acid metabolites and serum immune indicators. **(A)** Pearson correlation heatmap in the CON vs. SL group; **(B)** Pearson correlation heatmap in the CON vs. SH group; **(C)** Mediation test in the CON vs. SL group; **(D)** Mediation test in the CON vs. SH group; **(E)** Mechanistic model. *n* = 4 biological replicates selected from 6 lambs per group.

To further elucidate the mechanism by which SNG regulates host immunity through gut microbiota, we performed correlation and mediation analyses focusing on *Blautia*, key AA metabolites, and serum immune indices (Figure 8). Pearson correlation analysis revealed significant associations between *Blautia*, multiple AA metabolites, and serum cytokines across treatment groups (*p* < 0.05). Metabolites and immune indices were also strongly correlated, supporting the role of *Blautia* in coordinating metabolic and immune responses (Figures 8A, B). In the SL group, *Blautia* abundance was significantly negatively correlated with 15-Epiprostaglandin E1 and 1a,1b-Dihomo prostaglandin F_2_α (*p* < 0.05). Mediation analysis revealed that 15-Epiprostaglandin E1 and 1a,1b-Dihomo prostaglandin F_2_α completely mediated the effects of Blautia on IL-6 *(p* < 0.05). These findings indicate that low-dose SNG primarily modulates early immune responses through the *Blautia*–AA–immune axis. In the SH group, Blautia abundance correlated negatively with 15(S)-HpEPE and 20-hydroxy-LTB_4_ (*p* < 0.05), but positively with PG(4:0–16:0). Mediation analyses further revealed that 15(S)-HpEPE fully mediated the effects of *Blautia* on IFN-γ and IgG. Meanwhile, 20-hydroxy-LTB4 served as a complete mediator for IFN-γ and IL-4, and a partial mediator for IFN-γ and IL-6 (all *p* < 0.05).

Collectively, both SNG doses modulated *Blautia* abundance to influence AA metabolism and downstream immune activation. Low-dose SNG primarily initiated early immunomodulation, whereas high-dose SNG amplified AA-derived pro-inflammatory signaling, highlighting *Blautia* as a pivotal microbial bridge mediating SNG-induced immune enhancement in lambs (Figure 8).

## 4 Discussion

### 4.1 SNG promotes growth and enhances Th1-type immune activation in lambs

The present study demonstrated that dietary supplementation with SNG significantly increased the ADG of lambs, accompanied by marked alterations in serum immunoglobulins and cytokine profiles. These findings indicate that SNG has beneficial effects on both growth performance and immune modulation. The significant improvement in ADG observed in the SNG-treated lambs was consistent with previous reports showing that SNG supplementation enhanced ADG in piglets and broilers (15, 16). Notably, SNG supplementation elevated serum levels of IgG, IL-6, and IFN-γ, while reducing IL-4 and IL-10. Increased IgG suggests enhanced humoral immunity (17), and the shift toward Th1-type cytokines (IFN-γ, IL-6) alongside suppressed Th2-type cytokines (IL-4, IL-10) indicates a transition to a Th1-dominant cellular immune response (18). This immune phenotype transition represents one of the key findings of the current study. Traditionally, pro-inflammatory immune activation has been associated with growth suppression. However, in this study, the simultaneous elevation of Th1 cytokines (IFN-γ, IL-6) and ADG in SNG-supplemented lambs (19) challenges this classical paradigm. We propose that the Th1-skewed response induced by SNG does not indicate pathological inflammation, but rather a transition from a potentially energy-consuming “low-grade inflammation” or “immunosuppressive” state to a more efficient and controlled “active defense” state. IFN-γ can activate macrophage phagocytic and bactericidal functions (20), enabling faster clearance of potential pathogens and preventing chronic immune activation. Furthermore, Nakashima et al. (21) reported that dietary supplementation with *Euglena gracilis* significantly improved survival and accelerated viral clearance in H1N1-infected mice, with protective effects linked to increased pulmonary levels of IL-1β, IL-6, IL-12, and IFN-γ. This finding aligns with our results, jointly highlighting a crucial concept: moderate Th1-type immune activation driven by specific dietary bioactives is essential for establishing efficient protective immunity rather than necessarily causing growth inhibition. This mechanism aligns well with the “immuno-metabolic coupling” theory, which posits that maintaining a balanced immune state is critical to minimizing metabolic cost. By enhancing the efficiency and precision of immune responses, SNG may reduce the maintenance energy expenditure of the immune system, allowing more nutrients and energy to be redirected toward protein synthesis and muscle growth. Similarly, polysaccharides derived from *Bifidobacterium adolescentis* promote IFN-γ secretion via the TLR2/TLR4 pathway (22), and compound probiotics have also been reported to upregulate IFN-γ production (23). Together, these findings suggest that dietary regulation of Th1/Th2 balance represents a universal immune-modulatory mechanism. Therefore, SNG may optimize host immune status through modulation of gut microbiota or related signaling pathways, thereby improving animal health and productivity.

To note, significant imbalance in initial body weight existed among experimental groups despite random allocation, which may interfere with the interpretation of growth outcomes. Furthermore, we did not perform formal dose-trend statistical analysis in this trial; thus, linear dose-dependent effects of SNG extract cannot be confirmed. Further trials with balanced baseline body weight and gradient dosage design are required to verify linear dose-response relationships.

### 4.2 Oxidative stress regulation and its linkage to immune activation

Interestingly, although SNG triggered immune activation in lambs, both treatment groups exhibited significant reductions in T-AOC, SOD, and GSH-Px activities. Notably, the MDA level remained unchanged, indicating the absence of lipid peroxidation. While this apparent decline in antioxidant potential might seem counterintuitive, it likely reflects a strategic metabolic reallocation concomitant with immune activation rather than oxidative injury (24). During the early stages of an immune response, the controlled generation of reactive oxygen species (ROS) acts as signaling molecules that facilitate phagocytic bactericidal activity and cytokine signal transduction (25). Thus, transient consumption or downregulation of antioxidant enzymes may represent a physiological adaptation supporting effective immune priming. An analogous, yet fundamentally distinct, phenomenon has been observed in dairy cows exposed to deoxynivalenol (DON). In that context, DON functions as an exogenous toxin that induces excessive oxidative stress, and the subsequent elevation of antioxidant enzymes is interpreted as a passive compensatory response to oxidative damage (26). In contrast, the decline in antioxidant enzyme activities observed in SNG-treated lambs is more likely an active adaptive response, reflecting a redirection of metabolic flux toward immune cell activation and repair. Such “controlled oxidative activation” has been recognized as a hallmark of effective immune priming in recent immunometabolic studies (27). A moderate reduction in antioxidant buffering systems (e.g., GSH, SOD, GSH-Px) can enhance ROS-dependent signaling pathways, such as H_2_O_2_-mediated nuclear factor kappa B (NF-κB) activation, thereby lowering the activation threshold of macrophages and promoting Th1-type immune polarization (28, 29). Moreover, moderate ROS production supports macrophage polarization to the M1 phenotype and upregulates Th1-associated cytokines, including IFN-γ and IL-6 (30). Consequently, the reduced antioxidant enzyme activities in SNG-supplemented lambs may signify a coordinated immunometabolic reshaping aimed at optimizing immune efficiency rather than indicating oxidative stress. In summary, SNG's modulation of the antioxidant defense system appears to be part of a broader immunometabolic regulatory strategy. By transiently downregulating antioxidant enzyme activities, SNG may promote controlled ROS signaling, thereby enhancing Th1 cytokine production and efficient immune activation. This mechanism reinforces the emerging concept that mild oxidative activation, under well-regulated conditions, can serve as a physiological amplifier of immunity rather than a detrimental stressor. Although ROS levels were not directly measured here, the hypothesis of “controlled oxidative activation” merits further investigation.

### 4.3 Gut microbiota restructuring as a microbial foundation for immune modulation

The 16S rRNA sequencing analysis revealed that SNG supplementation markedly altered the composition of the lamb gut microbiota at the genus level. The relative abundances of *Blautia* and *Roseburia* were significantly decreased, whereas *Monoglobus* and *Colidextribacter* exhibited a dose-dependent increase. Combined LEfSe and random forest analyses further identified *Blautia* as the most sensitive and discriminative microbial biomarker in response to SNG treatment (Gini index = 3.174, AUC = 0.979). These alterations were not random but rather represent the core microbial basis underlying the immunomodulatory effects of SNG. As a key butyrate-producing genus in the intestinal ecosystem, the reduction of *Blautia* may constitute a critical step in SNG-mediated immune activation. Previous studies have demonstrated that *Blautia* can suppress Th1/Th17-type immune responses by producing metabolites such as butyrate and secondary bile acids, which inhibit dendritic cell activation (31, 32). Therefore, the decreased abundance of *Blautia* observed in this study may indicate a release of its immunosuppressive influence, thereby favoring the establishment of a Th1-dominant immune phenotype. In addition, immune activation is often accompanied by metabolic and redox shifts in the gut environment, which may create conditions unfavorable for the survival of strictly anaerobic taxa like *Blautia*, leading to its decline. This “functional de-repression” phenomenon suggests that the SNG-induced microbial restructuring contributes to promoting Th1-type immune responses. On the other hand, the enrichment of *Monoglobus* and *Colidextribacter* under SNG supplementation may further optimize host energy metabolism and the immunometabolic microenvironment. *Monoglobus* has been identified as a key genus involved in pectin degradation, and its enrichment reflects an enhanced bacterial capacity for dietary fiber fermentation (33). Meanwhile, *Colidextribacter* is closely associated with polysaccharide metabolism and SCFAs production, particularly propionate (34). Increased abundance of these genera could elevate SCFAs generation, providing additional energy substrates and participating in immune regulation—changes that may partly explain the improved ADG in SNG-treated lambs. Furthermore, functional prediction by PICRUSt2 provided strong metabolic evidence supporting the above microbial compositional shifts. SNG treatment significantly enriched pathways related to amino acid and carbohydrate metabolism, consistent with the metabolic functions associated with *Monoglobus* and *Colidextribacter*. Notably, sphingolipid metabolism and the phosphatidylinositol signaling system were significantly upregulated in the SH group, suggesting that SNG may modulate immune regulation through lipid signaling pathways. Sphingolipid metabolites, such as ceramide, are known to act as crucial mediators of inflammation and apoptosis (35, 36). Thus, SNG not only reshapes microbial composition but also reprograms the metabolic potential of the microbiota toward enhanced nutrient metabolism and immune signaling, providing a functional foundation for its immunomodulatory effects.

### 4.4 Metabolomic adaptation highlights AA–derived lipid mediators in immunoregulation

Untargeted metabolomic analysis revealed that SNG supplementation was associated with alterations in the fecal metabolic profiles of lambs. PCA and OPLS-DA analyses clearly differentiated the metabolic signatures of SNG-treated groups from those of the control, with the separation being most pronounced in the SH group. These findings indicate that SNG extract supplementation altered fecal metabolic characteristics differently under low and high inclusion levels. Notably, distinct AA–derived metabolite profiles were induced by different SNG doses, revealing a finely tuned immunoregulatory pattern. In the SH group, SNG was associated with the accumulation of 20-hydroxy-leukotriene B_4_, a bioactive metabolite closely related to leukotriene B_4_ (LTB_4_), which serves as a potent chemotactic and immune-activating lipid mediator (37, 38). The trend toward elevation of this metabolite may suggest that SNG at higher doses could influence immune cell recruitment and activation pathways, potentially contributing to the establishment of a Th1-dominant immune phenotype. Concurrently, the concentration of 15(S)-HpEPE—a precursor for pro-resolving mediators such as lipoxins (39)—also tended to increase, suggesting that SNG may simultaneously influence protective inflammation-resolving programs. This dual feature of “pro-inflammatory activation coupled with concurrent resolution” highlights an efficient, self-limiting immune defense strategy: the host mounts a rapid and effective Th1 response while concurrently preparing for inflammation resolution and tissue repair. In contrast, the SL group exhibited a distinct metabolic tendency toward the elevation of 15(R)-Lipoxin A_4_ and 15-Epiprostaglandin E1, both of which are classical inflammation-resolving lipid mediators. These molecules are known to inhibit neutrophil infiltration and promote macrophage-mediated clearance of apoptotic cells, thereby facilitating inflammation resolution and tissue restoration (40, 41). This pattern clearly indicates that low-dose SNG prioritizes maintaining immune homeostasis and promoting recovery rather than initiating a strong pro-inflammatory response. Collectively, these extract-inclusion-level-related lipidomic signatures suggest that SNG may elicit a balanced and context-specific immunometabolic adaptation, characterized by enhanced immune efficiency in the SH group and immune resolution support in the SL group.

### 4.5 Integrative microbiota–metabolite–immune axis reveals dose-dependent immunometabolic reprogramming

Further multi-omics integration revealed a “microbiota–metabolite–immune” regulatory axis centered on *Blautia*. Correlation analysis showed that *Blautia* abundance was significantly negatively associated with key AA–derived metabolites and pro-inflammatory cytokines, suggesting that its reduction may relieve intrinsic suppression of AA metabolism and immune activation (42, 43). Mediation analysis further confirmed that metabolites such as 15(S)-HpEPE, 20-hydroxy leukotriene B_4_, and 15-epiprostaglandin E1 acted as complete or partial mediators linking *Blautia* to downstream immune indicators (IL-6, IFN-γ, IgG), thereby establishing a causal “microbiota–metabolite–immune” interaction pathway.This regulatory axis exhibited a distinct dose-dependent bifurcation: in the SH group, *Blautia* inhibition was accompanied by the accumulation of Th1-associated metabolites (44, 45), reflecting an immune-activating phenotype; whereas in the SL group, *Blautia* interacted with 15-epiprostaglandin E1 to completely mediate its regulation of IL-6 (46, 47), suggesting a homeostatic and fine-tuned immune modulation pattern. Such a “microbiota–lipid mediator–immune” coupling mechanism is highly consistent with recent reports describing microbial metabolite–driven immune phenotype reprogramming (48).Collectively, SNG reshaped the *Blautia*–AA–immune axis in a dose-dependent manner, maintaining immune homeostasis at low doses while activating a protective Th1-polarized response at higher doses. This finding provides mechanistic insight into how SNG promotes synergistic enhancement of health and growth performance and offers a new theoretical framework for understanding how plant alkaloids can reprogram host “immune–metabolic” crosstalk via gut microbial remodeling (49).

## 5 Conclusion

In summary, this study demonstrates that dietary SNG extract enhances lamb growth performance and reshapes the gut microbiota–metabolite–immune regulatory axis to modulate host immune status, with divergent regulatory phenotypes observed under low and high extract supplementation levels. SNG supplementation reduced the abundance of *Blautia*, which in turn activated AA metabolism and promoted a Th1polarized cytokine response. The low-dose group (SL) primarily sustained immune homeostasis via anti-inflammatory lipid mediators, whereas the high-dose group (SH) induced a balanced increase in both pro-resolving and pro-inflammatory eicosanoids, supporting a protective yet controlled immune activation. Together, these results illustrate that SNG orchestrates a precise immunometabolic reprogramming that improves immune efficiency without provoking oxidative stress. Overall, our findings suggest a novel mechanism by which plant alkaloids can regulate host immunity and growth through microbial and metabolic networks, offering valuable insights for developing SNG-based functional feed additives to enhance health and productivity in ruminants.

## Data Availability

The 16S rRNA data presented in the study are deposited in the NCBI repository, accession number: PRJNA1510217, http://www.ncbi.nlm.nih.gov/bioproject/1510217/.
