# Supplementary material for: Exploring the impact of sanguinarine on lamb immunity based on gut microbiota and metabolomics
Source: Front Vet Sci. 2026 Sep 10;13:1926738. doi: 10.3389/fvets.2026.1926738 (PMC13600884; doi:10.3389/fvets.2026.1926738)
Supplement: Supplementary file 1 [file Table_1.DOCX]

Supplementary Material

# 1 Supplementary Tables

**Table S1.** Exact pairwise comparison *p*-values for serum biochemical and immune parameters (LSD post-hoc test)

| Parameter | CON vs. DIC | CON vs. SL | CON vs. SH | DIC vs. SL | DIC vs. SH | SL vs. SH |
| --- | --- | --- | --- | --- | --- | --- |
| T-AOC | 0.695 | <0.001 | <0.001 | <0.001 | <0.001 | 0.837 |
| SOD | 0.253 | <0.001 | 0.001 | <0.001 | <0.001 | 0.842 |
| GSH-Px | 0.115 | <0.001 | <0.001 | 0.003 | 0.01 | 0.547 |
| MDA | 0.03 | 0.782 | 0.223 | 0.05 | 0.261 | 0.338 |
| IgG | 0.47 | <0.001 | <0.001 | <0.001 | <0.001 | 0.439 |
| IL-4 | 0.002 | <0.001 | <0.001 | <0.001 | <0.001 | 0.274 |
| IL-6 | 0.007 | <0.001 | <0.001 | <0.001 | <0.001 | 0.647 |
| IL-10 | 0.015 | 0.039 | 0.006 | 0.615 | 0.599 | 0.311 |
| IL-1β | 0.888 | 0.469 | 0.293 | 0.39 | 0.237 | 0.73 |
| IFN-γ | 0.002 | <0.001 | <0.001 | <0.001 | <0.001 | 0.049 |
